# Perioperative safety and efficacy of robot-assisted total hip arthroplasty in ERAS-managed patients: a pilot study

**DOI:** 10.1186/s13018-023-04180-y

**Published:** 2023-09-18

**Authors:** Hanpeng Lu, Haocheng Sun, Qiang Xiao, Hong Xu, Qi Zhou, Linyuan Li, Tingfang Yan, Duan Wang, Zongke Zhou

**Affiliations:** 1 Department of Orthopaedic Surgery, West China Hospital, Sichuan University, No. 37, Guoxue Road, Wuhou District, Chengdu, 610041 Sichuan People’s Republic of China; 2 West China School of Medicine, Sichuan University, No. 37, Guoxue Road, Wuhou District, Chengdu, 610041 Sichuan People’s Republic of China; 3 Yuanhua Intelligent Technology (Shenzhen) Co., Ltd, Shenzhen, People’s Republic of China

**Keywords:** Robot-assisted total hip arthroplasty, ERAS, Blood loss, Pain, Functional recovery, Complications

## Abstract

**Aims:**

Robot-assisted total hip arthroplasty (rTHA) boasts superior accuracy in implant placement, but there is a lack of effective assessment in perioperative management in the context of enhanced recovery after surgery (ERAS). This study aimed to compare the effectiveness and safety of rTHA versus conventional total hip arthroplasty (cTHA) in ERAS-managed patients.

**Methods:**

In this prospective trial, a total of 60 eligible patients aged between 18 and 80 years were randomly divided into two groups to undergo either rTHA or cTHA. The primary outcomes included blood loss parameters. Secondary outcomes were the duration of the operation, surgical time, WOMAC pain score, WOMAC stiffness score, WOMAC physical function score, Harris score, and postoperative complications.

**Results:**

The study cohort analyzed 59 eligible participants, 30 of whom underwent rTHA and 29 of whom underwent cTHA. Analysis could not be conducted for one patient due to severe anemia. Notably, the cTHA group had a significantly shorter surgical time than the rTHA group (69.49 ± 18.97 vs. 104.20 ± 19.63 min, *P* < 0.001). No significant differences were observed between the rTHA and cTHA groups for blood loss parameters, including total blood loss (1280.30 ± 404.01 vs. 1094.86 ± 494.39 ml, *P* = 0.137) and drainage volume (154.35 ± 121.50 vs. 159.13 ± 135.04 ml, *P* = 0.900), as well as intraoperative blood loss (126.67 ± 38.80 vs. 118.52 ± 60.68 ml, *P* = 0.544) and hidden blood loss (982.43 ± 438.83 vs. 784.00 ± 580.96 ml, *P* = 0.206). Only one patient in the cTHA group required allogeneic blood transfusion. At 3 months postoperatively, both groups showed improvements in WOMAC pain score, WOMAC stiffness score, WOMAC physical function score, and Harris score, with no significant differences found between the two groups. Few complications were reported in both groups without significant differences.

**Conclusions:**

Despite the longer surgical time, rTHA did not negatively affect blood loss, pain, or functional recovery or lead to an increased risk of complications in ERAS-managed patients, suggesting that rTHA can be safely and effectively incorporated into an ERAS program for primary THA.

## Introduction

Total hip arthroplasty (THA) is widely considered one of the most successful surgical procedures in the twentieth century and a common approach in treating end-stage hip joint diseases [1]. However, as a major orthopedic surgery, THA typically involves a pathological, physiological, and metabolic decompensation process [2–4]. Enhanced recovery after surgery (ERAS) refers to a set of evidence-based perioperative optimization measures that aim to reduce physiological and psychological trauma, minimize postoperative complications, readmission, and mortality risks, promote accelerated postoperative rehabilitation, and shorten the length of stay (LOS) [5, 6]. Initially, applied in colon surgery [7], ERAS has gained increasing importance in orthopedic surgeries [8]. Decades of research have successfully controlled blood loss, improved functional outcomes, and reduced the length of hospital stay in THA patients [9].

Robot-assisted THA is a relatively new technology that has shown great clinical prospects [10] and can provide personalized, detailed preoperative planning, real-time intraoperative navigation, and soft tissue balancing [11]. Studies have compared robot-assisted total hip arthroplasty (rTHA) with conventional total hip arthroplasty (cTHA) and have found that rTHA has advantages in the accuracy of implant placement [12]. However, the relatively longer surgical time and additional registration procedures [13] may affect blood loss, postoperative pain, functional recovery, and complications. Therefore, it is meaningful to study whether rTHA will affect the fast recovery of ERAS-managed patients despite its validated merits.

In this prospective trial, we aimed to compare perioperative blood loss, pain, functional recovery, and complications between rTHA and cTHA. Through the analysis of these outcomes, we hope to determine the effectiveness and safety of robot-assisted THA in ERAS programs.

## Materials and methods

### Patients

**Fig. 1:**
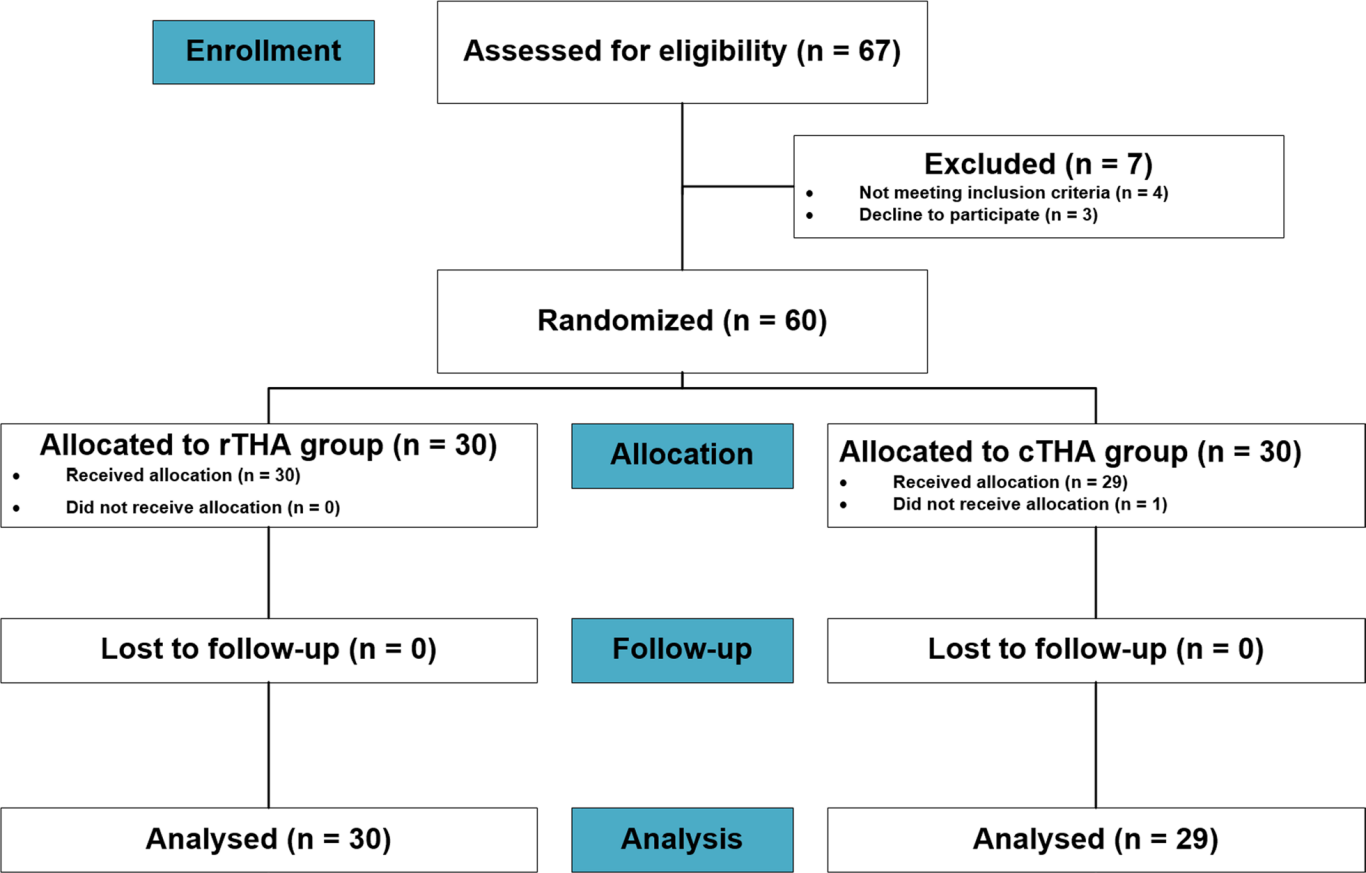
Consort (consolidated standards of reporting trials) flow diagram showing the process for incorporating participants through robotic arm-assisted total hip arthroplasty (rTHA) vs conventional total hip arthroplasty (cTHA)

The Ethics Committee on Clinical Trials of West China Hospital of Sichuan University approved this single-center, prospective trial (HX-IRB-AF-12-V4.0) involving participants aged 18–80 years. All patients provided written informed consent. Exclusion criteria included neuromuscular dysfunction, active infection lesions, severe hip deformity, hip dysplasia with Crowe grade 3 or 4, ankylosing spondylitis patients with bony ankylosis or severe stiffness, bilateral hip arthroplasty, severe internal and surgical diseases, weak physique, and poor expected compliance. A total of 67 patients were scheduled to undergo primary THA, 4 were ineligible, and 3 declined participation (Fig. 1). The remaining 60 patients were randomly divided into two groups by using the central stochastic system and interactive web response system.

### Sample size calculation

This study was designed as a randomized controlled trial to assess non-inferiority. The experimental group consisted of patients undergoing rTHA, while the control group comprised patients undergoing cTHA. The primary outcome measure was the total amount of bleeding in the study participants. Based on the findings from our preliminary experiment, there was a difference of 115 between the experimental and control groups. With a bilateral α level of 0.05 and a power (1 − *β*) of 0.8, the non-inferiority margin was set at 179. The sample size ratio between the experimental and control groups was 1:1. Following the approach outlined by Chow et al. [14], sample size calculations using the R programming language yielded a required sample size of 28 cases in each group. Consequently, a total of 60 cases were included in this study.

### Surgical procedure and perioperative management

Patients underwent either conventional THA or robotic arm-assisted THA. The rTHA was operated with a single semiactive surgical robot (YUANHUA-THA) whose surgical procedures were described in the technical manuals provided by the manufacturer. All patients were administered the standard ERAS protocol in our hospital including tranexamic acid (TXA) for blood loss prevention, pain management techniques, thrombosis prevention measures, and functional rehabilitation. Specifically, patients were prescribed a twice-daily dose of 200 mg celecoxib from admission until 14 days after discharge. As part of our TXA protocol, patients received 2 g of intravenous TXA 30 min before the incision, followed by 1 g of intravenous TXA at 3 and 6 h after the surgery. Prior to TXA administration, all patients received 5 mg of dexamethasone intravenously. For thrombosis prevention, low molecular weight heparin (enoxaparin) in 0.2 ml was administered 8 h after the surgery and increased to 0.4 ml per day on subsequent days until discharge. Patients were then prescribed apixaban for two weeks.

### Clinical outcomes

The primary outcome was assessed by measuring various parameters related to blood loss, as blood management was a very crucial component of ERAS. Objective indicators such as total blood loss, intraoperative blood loss, drainage volume, blood transfusion volume, and hidden blood loss were measured. The theoretical bleeding volume of each patient was determined by Gross's formula [15]. The total blood loss is equal to the theoretical bleeding amount plus the transfusion amount. Intraoperative blood loss was determined by estimating the weight increase of gauze and the volume (excluding saline) in the negative pressure aspirator bottle. The drainage volume was recorded upon removal of the drainage tube 24 h following the procedure. The postoperative blood loss was estimated by considering the drainage volume and dressing weight, while the hidden blood loss was calculated by subtracting the intraoperative and postoperative blood loss from total blood loss.

The secondary outcomes were measured based on several parameters, such as the duration of the operation, surgical time, WOMAC score, Harris score, and postoperative complications. These outcomes were evaluated on admission, every day from postoperative days (PODs) 1–3, the day of discharge, and one and three months after surgery. Postsurgical complications, such as anemia, hypoproteinemia, hypokalemia, arthralgia, superficial infection, dysuria, numbness of lower limbs, dislocation, deep vein thrombosis, and pulmonary embolism, were monitored.

### Statistical analysis

Means and standard deviations were used for quantitative variables, and percentages were used for qualitative variables. Independent sample *t* tests were utilized for comparisons. For continuous data with a normal distribution, an independent-samples *t* test was employed, while the Mann‒Whitney *U* test was used for continuous data with skew. Categorical data were analyzed using either the Chi-squared test or Fisher's precision test. All data analyses were completed using SPSS 27.0 (SPSS Inc., Chicago, IL, USA). Statistical significance was set at *P* < 0.05.

## Results

All patients were enrolled between April 2022 and June 2022 at West China Hospital, Sichuan University, and were randomly assigned to two groups. Of the 60 initially eligible patients treated during the recruitment period, only 1 was excluded due to severe anemia. The final study cohort consisted of 30 patients with rTHA and 29 with cTHA. Both groups showed no significant differences in demographic characteristics (Table 1). Notably, cTHA had a significantly shorter surgical time than rTHA (69.49 ± 18.97 vs. 104.20 ± 19.63 min, *P* < 0.001). Hospital days showed no significant difference between the two groups, with 5.29 ± 0.53 days in rTHA versus 5.31 ± 0.54 days in cTHA (*P* = 0.863).

**Table 1:** Preoperative demographic data of analyzed patients

	rTHA (*n* = 30)	cTHA (*n* = 29)	*P* value
*N*	30	29	
Age	56.00 ± 12.33	56.52 ± 11.93	0.871
Sex, *n* (%)			0.329
Male	13 (43.33)	9 (31.03)	
Female	17 (56.67)	20 (68.97)	
Surgical side, *n* (%)			0.902
Left	16 (53.33)	15 (51.72)	
Right	14 (46.67)	14 (48.28)	
BMI (kg/m^2^)	24.26 ± 3.39	22.62 ± 3.12	0.058
Comorbidities, *n* (%)			
Pulmonary disease	5 (16.67)	8 (27.59)	0.312
Hypertension	7 (23.33)	6 (20.69)	0.807
Chronic kidney disease	2 (6.67)	6 (20.69)	0.145
Diabetes	1 (3.33)	2 (6.90)	0.612
Cardiovascular disease	5 (16.67)	2 (6.90)	0.424
Liver disease	8 (26.67)	4 (13.79)	0.219

rTHA, robot-assisted total hip arthroplasty; cTHA, conventional total hip arthroplasty; BMI, body mass index

**Fig. 2:**
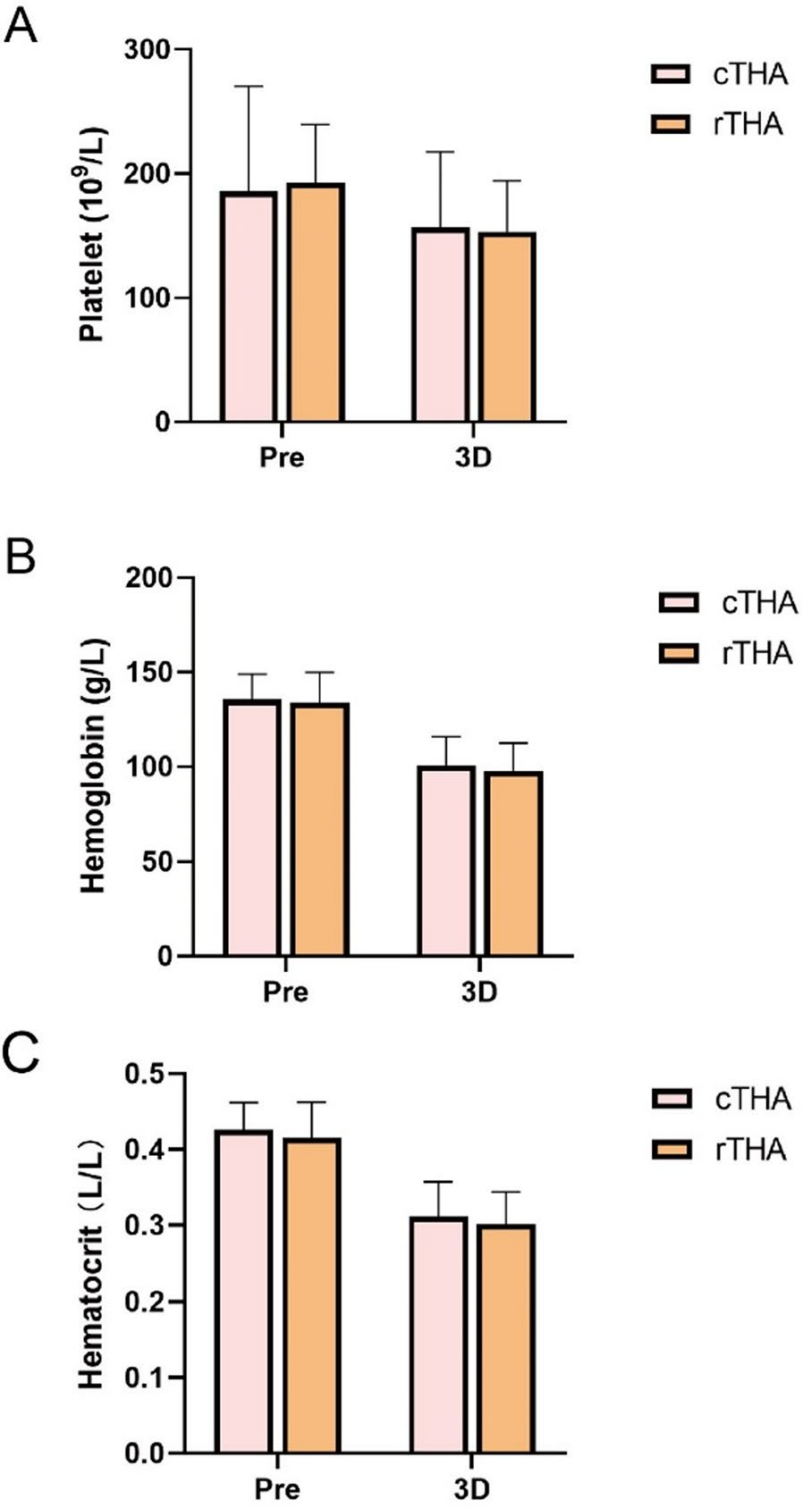
Perioperative blood indicators of hemoglobin concentration (**A**), hematocrit (**B**), and platelet (PLT) count (**C**). Pre, preoperative within 14 days; 3D, postoperative 3 days

Regarding blood loss parameters, there was no significant difference in total blood loss (1280.30 ± 404.01 vs. 1094.86 ± 494.39 ml, *P* = 0.137) or drainage volume (154.35 ± 121.50 vs. 159.13 ± 135.04 ml, *P* = 0.900) between the rTHA and cTHA groups. Additionally, there were no significant differences in intraoperative blood loss (126.67 ± 38.80 vs. 118.52 ± 60.68 ml, *P* = 0.544) or hidden blood loss (982.43 ± 438.83 vs. 784.00 ± 580.96 ml, *P* = 0.206) between the two groups. Only one patient in the cTHA group required blood transfusion (Table 2). Table 3 shows the preoperative and postoperative (3 days) hemoglobin concentration, hematocrit, and platelet count changes with no significant differences observed between the groups. Figure 2 provides a visual representation of these changes.

**Table 2:** Perioperative status in the two patient groups

Variable	rTHA (*n* = 30)	cTHA (*n* = 29)	*P* value
Surgical time (min)	104.20 ± 19.63	69.49 ± 18.97	< 0.001^*^
Hospital stay (day)	5.29 ± 0.53	5.31 ± 0.54	0.863
Total blood loss (ml)	1280.30 ± 404.01	1094.86 ± 494.39	0.137
Intraoperative blood loss (ml)	126.67 ± 38.80	118.52 ± 60.68	0.544
Drainage volume (ml)	154.35 ± 121.50	159.13 ± 135.04	0.900
Hidden blood loss (ml)	982.43 ± 438.83	784.00 ± 580.96	0.206
Transfusion, *n* (%)	0 (0.00)	1 (3.4)	0.492

^*^Represents *P* < 0.05

**Table 3:** Perioperative blood indicators of hemoglobin concentration, hematocrit, and platelet count

	cTHA (*n* = 30)	rTHA (*n* = 29)	*P* value
Platelet (10^9^/L)			
Pre	185.72 ± 84.72	192.70 ± 47.02	0.696
POD 3	156.45 ± 61.06	153.03 ± 41.01	0.801
Hemoglobin (g/L)			
Pre	135.66 ± 13.27	133.80 ± 16.04	0.631
POD 3	100.34 ± 15.52	97.50 ± 15.00	0.477
Hematocrit (L/L)			
Pre	0.43 ± 0.04	0.42 ± 0.05	0.359
POD 3	0.31 ± 0.05	0.30 ± 0.04	0.368

Improvements in WOMAC pain score, WOMAC stiffness score, WOMAC physical function score, and Harris score were observed at 3 months postoperatively compared to preoperative values, with no significant differences found among the groups (Table 4). Few complications were reported for both groups without significant differences (Table 5). Specifically, 11 patients in the two groups experienced postoperative anemia, with 1 patient in the rTHA group and 5 patients in the cTHA group showing hypoproteinemia. One case of hypokalemia and arthralgia was reported in cTHA. In rTHA, 2 cases of dysuria, 1 case of numbness in the lower limbs. No pulmonary embolism or lower extremity deep vein thrombosis was reported in either group.

**Table 4:** Preoperative functional status and postoperative functional outcomes

	rTHA (*n* = 30)	cTHA (*n* = 29)	*P* value
Harris score			
Preoperative	53.55 ± 13.93	54.71 ± 9.52	0.718
3 months	96.81 ± 5.15	97.23 ± 4.26	0.740
WOMAC score			
Preoperative (total)	47.07 ± 13.71	45.89 ± 10.54	0.889
Pain	9.63 ± 2.81	9.56 ± 2.41	1.000
Stiffness	2.53 ± 1.43	2.67 ± 1.14	0.565
Physical function	34.93 ± 10.28	33.67 ± 7.80	0.625
3 months (total)	4.30 ± 4.54	4.00 ± 4.27	0.875
Pain	0.30 ± 0.60	0.33 ± 1.04	1.000
Stiffness	0.53 ± 0.73	0.56 ± 0.75	0.612
Physical function	3.47 ± 3.60	3.11 ± 3.12	0.657

The WOMAC score includes three aspects of evaluation: pain, stiffness, and physical function in daily life

**Table 5:** Postoperative complications

Variable, No. (%)	rTHA (*n* = 30)	cTHA (*n* = 29)	*P* value
Anemia	11 (36.67)	11 (37.93)	0.920
Hypoproteinemia	1 (3.33)	5 (17.24)	0.181
Hypokalemia	0 (0.00)	1 (3.45)	0.492
Arthralgia	0 (0.00)	1 (3.45)	0.492
Superficial infection	0 (0.00)	0 (0.00)	–
Dysuria	2 (6.67)	0 (0.00)	0.492
Numbness of lower limbs	1 (3.33)	0 (0.00)	1.000
Deep vein thrombosis	0 (0.00)	0 (0.00)	–
Pulmonary embolism	0 (0.00)	0 (0.00)	–

## Discussion

Theoretically, the longer operation time was negative on blood loss, postoperative pain and functional recovery in rTHA due to increased tissue trauma, fluid shifts and swelling, and prolonged exposure to anesthesia. However, the results of our study showed that rTHA did not have a negative impact on fast recovery in ERAS-managed patients compared to conventional THA. Both groups had comparable outcomes in terms of blood loss, functional recovery, and complications.

rTHA requires additional registration procedures and has a longer surgical time than cTHA, which are obvious defects[16]. Our study found that although cTHA had a significantly shorter surgical time than rTHA, there were no significant differences in blood loss parameters, such as total blood loss, drainage volume, intraoperative blood loss, or hidden blood loss. Similarly, no significant differences were observed in hospital days or hemoglobin concentration changes between the two groups. Patient blood management is a crucial technology in reducing the need for blood transfusion in orthopedic surgery [17]. Despite the significant risk of bone surface bleeding in orthopedic surgery, the application of controlled hypotension and antifibrinolytic drugs can significantly reduce bleeding and lower transfusion rates [18, 19]. Furthermore, orthopedic surgery is a high-risk surgery for venous thromboembolism, and achieving a balance between hemostasis and anticoagulation remains a core scientific issue. At our center, we routinely perform hemostasis and antifibrinolysis after THA, and the ERAS program is effective in cTHA [20]. In this study, we promptly used tranexamic acid perioperatively and low molecular weight heparin and apixaban for anticoagulation after surgery followed the same ERAS protocol without intergroup differences. Although rTHA patients had a longer operation time, this extra time did not increase the consumption of tranexamic acid or incur additional intervention and/or pharmaceutical costs. We observed no significant differences in bleeding between the rTHA and cTHA groups, which demonstrates that even with longer surgical time and incisions in the rTHA group, our ERAS blood management protocol effectively managed bleeding, and therefore, we can still manage bleeding effectively using our ERAS blood management plan in rTHA.

Both the rTHA and cTHA groups showed significant improvements in WOMAC and Harris score at 3 months postoperatively compared to preoperative values. Shibanuma et al. reported that the HHS at discharge was significantly higher in the rTHA group than in the cTHA group [21]. Similarly, a few studies have reported reduced pain, increased patient satisfaction, and improved functional outcomes as assessed using the HHS in rTHA [22, 23]. These studies have compared the outcomes of rTHA and cTHA, showing better functional outcomes. These outcomes confirm that the longer surgical time of rTHA did not negatively affect functional recovery compared to cTHA in short-term follow-up. Moreover, although an increased surgical duration may evoke a stronger physiological stress response, it is possible that this response is transient and does not impact the patients' functionality after the three-month postoperative period. Therefore, we did not observe any differences in functional scores between the two groups. Additionally, while the early postoperative functional recovery between rTHA and cTHA shows no significant differences, further follow-up is necessary to determine whether there are any differences in long-term outcomes.

Complications are always a concern in any surgical procedure. Our study found a low incidence of complications in both the rTHA and cTHA groups, with no significant differences between the two groups. Although some patients in the rTHA group experienced dysuria, and numbness in the lower limb, these differences were not statistically significant. Previous meta-analyses have shown that rTHA has better radiographic outcomes, including a higher incidence of safe zones in Lewinnek and Callanan [24]. Superior implantation can reduce the risk of mechanical failure of prosthetic joints, which means that rTHA can reduce the risk of complications. However, in most studies, patients receiving rTHA had longer surgical times [25]. These findings suggest that rTHA may have a higher risk of perioperative complications, especially during the learning curve associated with rTHA, as the prolonged surgical time in rTHA is associated with an increased risk of adverse outcomes [26, 27]. This also explains why there was 1 case of numbness in the lower limbs in rTHA.

Our study has several limitations. First, our sample size was relatively small. Therefore, future studies should consider including more cases. However, our analysis found that the sample size was sufficient for the primary outcomes. Second, our study had a short follow-up time, as it was intended to verify whether rTHA affects ERAS rehabilitation. Further research is needed to confirm these findings and evaluate the long-term outcomes of rTHA.

In conclusion, our study found that rTHA does not negatively impact fast recovery in ERAS-managed patients and is associated with comparable outcomes in terms of blood loss, pain, functional recovery, and complications when compared to cTHA. Therefore, rTHA can be considered a safe and effective option for hip arthroplasty in ERAS-managed patients.

## References

[1] Zagra L (2017). Advances in hip arthroplasty surgery: What is justified?. Efort Open Reviews.

[2] Carli F (2015). Physiologic considerations of Enhanced Recovery After Surgery (ERAS) programs: implications of the stress response. Can J Anaesth.

[3] Kelliher LJS, Scott M (2022). Modifying the stress response—Perioperative considerations and controversies. Anesthesiol Clin.

[4] Zhu S, Qian W, Jiang C, Ye C, Chen X (2017). Enhanced recovery after surgery for hip and knee arthroplasty: a systematic review and meta-analysis. Postgrad Med J.

[5] Brodner G, Pogatzki E, Van Aken H, Buerkle H, Goeters C, Schulzki C, Nottberg H, Mertes N (1998). A multimodal approach to control postoperative pathophysiology and rehabilitation in patients undergoing abdominothoracic esophagectomy. Anesth Analg.

[6] Wainwright TW (2020). Consensus statement for perioperative care in total hip replacement and total knee replacement surgery: Enhanced Recovery After Surgery (ERAS(R)) Society recommendations. Acta Orthop.

[7] Cavallaro P, Bordeianou L (2019). Implementation of an ERAS pathway in colorectal surgery. Clin Colon Rectal Surg.

[8] Soffin EM, YaDeau JT (2016). Enhanced recovery after surgery for primary hip and knee arthroplasty: a review of the evidence. Brit J Anaesth.

[9] Morrell AT, Layon DR, Scott MJ, Kates SL, Golladay GJ, Patel NK (2021). Enhanced recovery after primary total hip and knee arthroplasty a systematic review. J Bone Joint Surg-Am.

[10] Wright-Chisem J, Elbuluk AM, Mayman DJ, Jerabek SA, Sculco PK, Vigdorchik JM (2022). The journey to preventing dislocation after total hip arthroplasty: how did we get here?. Bone Joint J.

[11] Sweet MC, Borrelli GJ, Manawar SS, Miladore N (2021). Comparison of outcomes after robotic-assisted or conventional total hip arthroplasty at a minimum 2-year follow-up: a systematic review. JBJS Rev.

[12] Ng N, Gaston P, Simpson PM, Macpherson GJ, Patton JT, Clement ND (2021). Robotic arm-assisted versus manual total hip arthroplasty A SYSTEMATIC REVIEW AND META-ANALYSIS. Bone Joint J.

[13] Bullock EKC, Brown MJ, Clark G, Plant JGA, Blakeney WG (2022). Robotics in total hip arthroplasty: current concepts. J Clin Med.

[14] Chow SC, Tu YH (2008). On srials. J Formos Med Assoc.

[15] Gross JB (1983). Estimating allowable blood loss: corrected for dilution. Anesthesiology.

[16] Chen X, Xiong J, Wang P, Zhu S, Qi W, Peng H, Yu L, Qian W (2018). Robotic-assisted compared with conventional total hip arthroplasty: systematic review and meta-analysis. Postgrad Med J.

[17] Pennestrì F, Maffulli N, Sirtori P, Perazzo P, Negrini F, Banfi G, Peretti GM (2019). Blood management in fast-track orthopedic surgery: an evidence-based narrative review. J Orthop Surg Res.

[18] Memtsoudis SG, Fiasconaro M, Soffin EM, Liu JB, Wilson LA, Poeran J, Bekeris J, Kehlet H (2020). Enhanced recovery after surgery components and perioperative outcomes: a nationwide observational study. Brit J Anaesth.

[19] Ma J, Huang ZY, Shen B, Pei FX (2014). Blood management of staged bilateral total knee arthroplasty in a single hospitalization period. J Orthopaedic Surg Res.

[20] Luo ZY, Wang D, Meng WK, Wang HY, Pan H, Pei FX, Zhou ZK (2018). Oral tranexamic acid is equivalent to topical tranexamic acid without drainage in primary total hip arthroplasty: a double-blind randomized clinical trial. Thromb Res.

[21] Shibanuma N, Ishida K, Matsumoto T, Takayama K, Sanada Y, Kurosaka M, Kuroda R, Hayashi S (2021). Early postoperative clinical recovery of robotic arm-assisted vs. image-based navigated Total hip Arthroplasty. BMC Musculoskelet Disord.

[22] Perets I, Walsh JP, Close MR, Mu BH, Yuen LC, Domb BG (2018). Robot-assisted total hip arthroplasty: clinical outcomes and complication rate. Int J Med Robot.

[23] Cordero-Ampuero J, de Dios M (2010). What are the risk factors for infection in hemiarthroplasties and total hip arthroplasties?. Clin Orthop Relat Res.

[24] Ulrich SD, Seyler TM, Bennett D, Delanois RE, Saleh KJ, Thongtrangan I, Kuskowski M, Cheng EY, Sharkey PF, Parvizi J (2008). Total hip arthroplasties: What are the reasons for revision?. Int Orthop.

[25] Singh V, Realyvasquez J, Simcox T, Rozell JC, Schwarzkopf R, Davidovitch RI (2021). Robotics versus navigation versus conventional total hip arthroplasty: Does the use of technology yield superior outcomes?. J Arthroplasty.

[26] DeFrance MJ, Yayac MF, Courtney PM, Squire MW (2021). The impact of author financial conflicts on robotic-assisted joint arthroplasty research. J Arthroplasty.

[27] O'Malley NT, Fleming FJ, Gunzler DD, Messing SP, Kates SL (2012). Factors independently associated with complications and length of stay after hip arthroplasty analysis of the national surgical quality improvement program. J Arthroplasty.

